# Wild bee diversity of the National Park of the Semois Valley (Belgium)

**DOI:** 10.3897/BDJ.13.e144223

**Published:** 2025-02-12

**Authors:** Maxence Gérard, William Fiordaliso, Louise Ferrais, Chloé Fournier, Malo Hairault, Lise Lheureux, Paolo Rosa, Guillaume Ghisbain

**Affiliations:** 1 Laboratory of Zoology, Research Institute for Biosciences, University of Mons, Place du parc 20, Mons, Belgium Laboratory of Zoology, Research Institute for Biosciences, University of Mons, Place du parc 20 Mons Belgium; 2 Laboratory of Interaction Ecology and Global Change, Research Institute for Biosciences, Mons, Belgium Laboratory of Interaction Ecology and Global Change, Research Institute for Biosciences Mons Belgium; 3 Parc National de la Vallée de la Semois, Parc Naturel de Gaume, Rue Camille Joset 1, Rossignol, Belgium Parc National de la Vallée de la Semois, Parc Naturel de Gaume, Rue Camille Joset 1 Rossignol Belgium

**Keywords:** Ardenne, forest, inventory, pollinator, Red List

## Abstract

**Background:**

Wild bees are essential pollinators, yet their decline due to human activities threatens ecosystem stability. Protecting these pollinators requires a detailed understanding of both their diversity and distribution. In Belgium, the recently-established Semois Valley National Park (SVNP) is located in a region with limited bee sampling data and this study aims to identify the habitats most suitable to bees, especially for threatened species.

**New information:**

Over five months, we surveyed 32 sites and collected a total of 1,119 specimens belonging to 120 bee species. Twenty-two of the observed species are listed as threatened in Belgium according to the last Red List published in 2019 for the country, four of them being Critically Endangered. Our findings indicate that mesic grasslands support the highest species diversity, as well as the highest number of threatened species. Our results underscore the need for conservation efforts aimed at maintaining diversity and species richness in this region. Effective biodiversity preservation will require enhanced habitat management and strategies tailored to bee species' ecological requirements.

## Introduction

**Figure 1.**
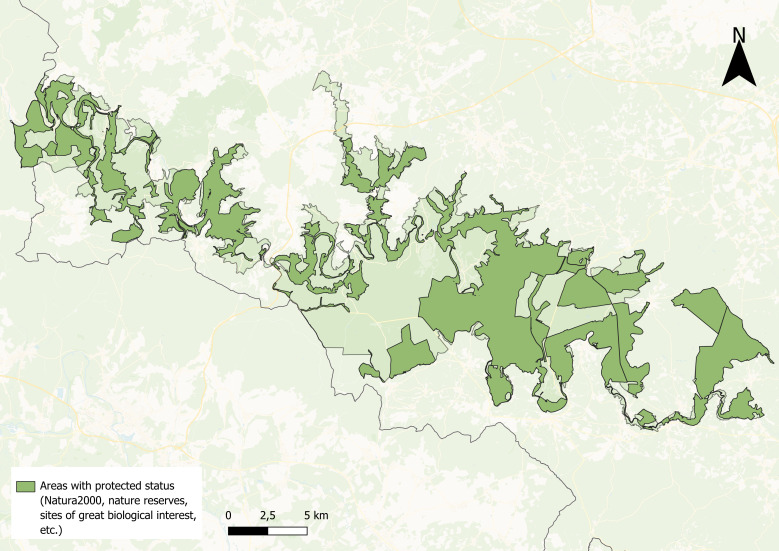
Distribution of protected areas located in the Semois Valley National Park, Belgium, including Natura 2000 sites, sites of great biological interest (SGIB), nature reserves and wetlands of biological interest (ZHIB).

Given the current rate of biodiversity loss, conservation must be a global priority for governments and organisations to safeguard ecosystems functioning and ensure a sustainable future (Diaz 2019). In this context, a call for projects was launched to establish the first two National Parks in Wallonia, Belgium. Amongst these, the Semois Valley was officially designated a National Park on 9 December 2022. The Semois Valley National Park (SVNP) spans 28,903 hectares across the provinces of Luxembourg and Namur. Renowned for its dense forests and network of rivers, the Park is primarily centred around the Semois River, with forests covering 86.54% of its area. The Park contains a rich diversity of habitats categorised using the European Nature Information System (EUNIS), including mesic grasslands (E2 - 9% of the NP area), seasonally wet and wet grasslands (E3 - 0.7% of the NP area) and riverine and fen scrubs (F9 - 0.3% of the NP area). Additionally, the SVNP features unique habitats shaped by historical human activity, such as inactive slate quarries from the 19^th^ century, which were once part of the region’s slate industry. These former industrial activities, once economically significant for Wallonia, now contribute to shaping the Park's landscape (Remacle 2007). The Park is also subject to various protection designations that cover 15,648 hectares (ca. 54% of its total area; Fig. 1) and includes 10 Natura 2000 sites. Moreover, it contains four biologically important wetlands, totalling 3.10 hectares (Parc National de la Vallée de la Semois 2021). The extensive protection statuses across a large portion of the Park underscore a strong regional commitment to preserving the landscape and its biodiversity.

Bee diversity in Belgium is represented by 419 species (389 species of them having recent occurrence records - Vertommen et al. (2024)), which corresponds to ca. 20% of the European diversity (Ghisbain 2023, Reverté et al. 2023). In some regions, over a century of field sampling has provided a detailed overview of species diversity and insights into population trends (Rasmont 1988, Rasmont 2005, Drossart 2019, Vray et al. 2019, Rollin et al. 2020, Schatz et al. 2021). The role of bees in Belgium's crop production, which is dependent on pollinator activity, is critical and is estimated to contribute to €251.6 million in 2010, including €0.59 million for Luxembourg province (Jacquemin et al. 2017). More broadly, between 78% and 90% of global pollination of flowering plants is performed by animals (Ollerton et al. 2011, Tong et al. 2023). Pollinators therefore play a critical functional role in most terrestrial ecosystems and agroecosystems in maintaining both wild plant communities and ensuring agricultural productivity (Potts et al. 2010). However, patterns of decline of wild bee populations have been repeatedly observed in the last decades (Biesmeijer et al. 2006, Nieto 2014, Zattara and Aizen 2021, Ghisbain et al. 2024), with five anthropogenic factors contributing to this phenomenon: intensive pesticide use, land-use and management changes, climate change, interspecific competition with massively introduced managed species and the impact of pests and pathogens (Potts et al. 2010, Goulson et al. 2015, Geslin et al. 2017, Dicks et al. 2021, Ghisbain et al. 2021a). In Belgium, 45 wild bee species are considered “Regionally Extinct” (although some were later rediscovered by Vertommen et al. (2024)), while 113 are classified as threatened according to the Belgian Red List of bees (Drossart 2019). Based on citizen-science databases (Parc National de la Vallée de la Semois 2021), the habitats within the Semois Valley National Park (SVNP) support 188 bee species, representing nearly half of Belgium’s bee diversity, making it a particularly valuable region for study.

To address the challenge of mitigating wild bee decline, conservation initiatives, such as the establishment of National Parks, coupled with standardised monitoring protocols, are essential (Drossart and Gérard 2020). Unfortunately, previous scientific inventories in Wallonia have been limited and did not always adhere to standardised methods. Furthermore, certain regions, such as the Semois Valley National Park (SVNP), have been notably under-sampled, both before and after 1970, as indicated by the occurrence maps published in the Red List of Belgian Bees of Drossart (2019). While participatory citizen-science platforms (https://observations.be/) provide useful and interesting insights into the diversity of wild bees within the SVNP, these data are not systematically verified (Turley et al. 2024). Moreover, although some naturalist surveys have been conducted in a few of the Park’s communes, no scientific study has yet proposed a protocol that focuses on associating wild bee species to specific habitats. In this context, the primary goal of this preliminary monitoring is to contribute to the characterisation of these under-studied bee populations and will help meet the long-term strategic and operational objectives of the SVNP. Indeed, accurate knowledge about the local fauna is crucial for guiding future biodiversity conservation policies within the Park.

In this report, we present the observations made during a standardised wild bee inventory conducted in spring and summer 2024 across various habitat types within the SVNP. We also emphasise the importance of certain habitats for several noteworthy bee species that are either classified as threatened on the Belgian Red List of bees or are legally protected in Belgium, while enhancing understanding of the habitats associated with them.

## Materials and methods

**Selection of sampling sites**

**Figure 2.**
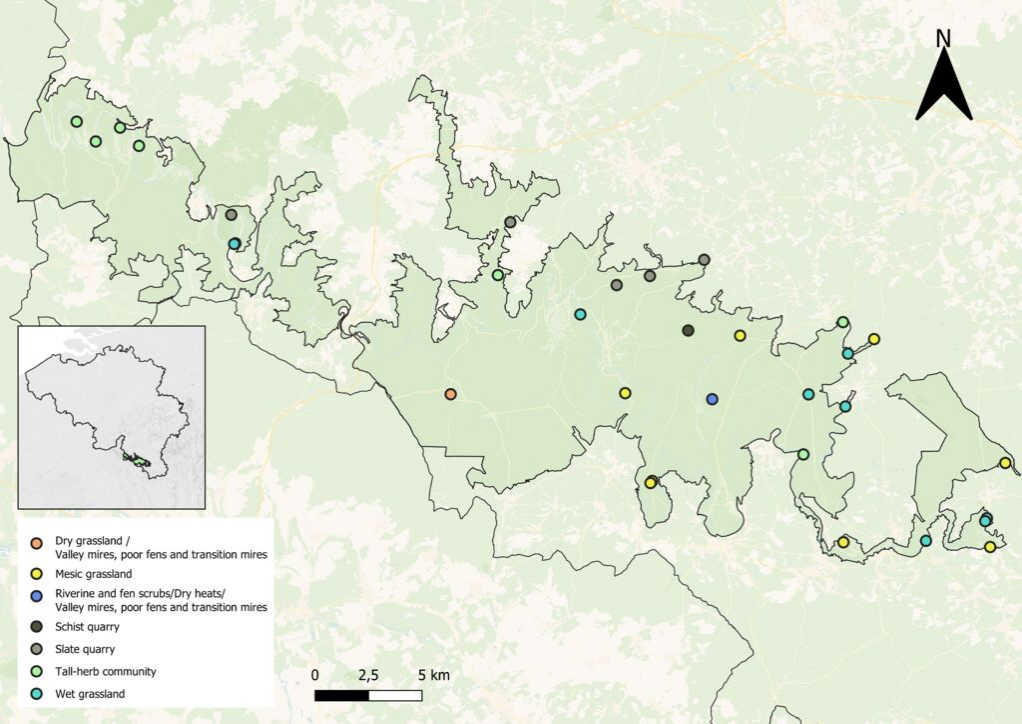
Distribution of sampling sites. The 32 sites are located in the Semois Valley National Park, Belgium. The colour code represents the habitat type at each sampling site.

The selection of sites aimed to ensure representativeness across habitats to best estimate the diversity of wild bees. A total of 32 sites were chosen, based on their expected diversity of plants and bees. These sites encompass a wide range of habitats within the SVNP (Fig. 2), most of which are classified using EUNIS codes. Amongst the habitats particularly favourable to bees, **mesic grasslands** (EUNIS E2) are mown twice a year. These grasslands are characterised by light to moderate fertilisation and generally support a diverse range of plant species adapted to moderately fertile soils. Additionally, due to the presence of the Semois River, numerous wet habitats are found within the SVNP. **Seasonally wet and wet grasslands** (E3) are ecosystems where water is present seasonally or even permanently. These areas are typically mown once or twice a year and develop on mesotrophic soils, which are relatively rich in minerals and nitrogen. **Tall-herb communities of humid meadows** (E5.42) are distinguished by spongy, moist soils resulting from the accumulation of organic matter. These habitats often emerge due to the abandonment of traditional agricultural practices, such as grazing and mowing, allowing for dense growth of large-leaved plants. As a result, they represent a successional stage that wet meadows may progress towards. **Valley mires, poor fens and transition mires** (D2) form in depressions, gentle slopes and areas waterlogged by rising water tables. Another wet habitat, **riverine and fen scrubs** (F9), represents near-climax formations, which are more stable in particularly wet sites with a high water table near the surface. In contrast to these wet habitats, a few drier habitats remain in the SVNP, though they are geographically restricted. Amongst these, **dry grasslands** (E1) are typically low-growing and result from past agro-pastoral practices. Today, only a few parcels in the SVNP have not been impacted by either reforestation or intensive agriculture. **Dry heaths** (F4.2) are characterised by siliceous soils, with open areas covered by mosses or lichens and plant species from the Ericaceae family. The region's soil was also exploited for its mineral content, with **schist quarries** historically used to extract materials for road construction, as well as the aforementioned **slate quarries**. These last two types of habitats are not associated with any EUNIS code.

In total, eight sites of mesic grassland, eight sites of wet grassland and eight sites of tall-herb communities in humid meadows were selected. Additionally, five slate quarries, one schist quarry, one site combining mires, dry heaths and riverine scrubs, and one site combining dry grasslands and mires were selected. To maintain consistency in our sampling protocol and, due to the insufficient presence of the latter habitats in the National Park to include eight sites per habitat type, these last eight sites were grouped under the category 'other habitats' for the purposes of presenting the results in figures. Although ecologically heterogeneous, these eight additional sites aim to provide a comprehensive overview of bee diversity within the SVNP.

All sites were spaced at least one kilometre apart to minimise spatial autocorrelation during the inventories, ensuring that individuals collected from different sites were unlikely to be present in multiple locations, thus avoiding double sampling effort (Gathmann and Tscharntke 2002). The sampled sites were identified by the SVNP project leaders and employees. Private landowners of the selected sites were contacted and informed in advance. Additionally, forest rangers from the Département Nature et Forêt (DNF), responsible for safeguarding state and public land, were consulted. For each sampled site, an official collection permit has been delivered by the Service Public de Wallonie (SPW).

**Sampling protocol**

Throughout the study, each of the 32 sites was sampled monthly from April to August, resulting in a total of five sampling sessions per site across the entire inventory period. These months coincide with the majority of the active season for wild bees in Belgium (Duchenne et al. 2020). To ensure data consistency, a standardised sampling protocol was followed. Each sampling session involved 40 minutes of effective net collection time, which refers to the time spent actively searching for individuals within the site. The size of the sites ranged from 0.15 ha to 12.39 ha (mean = 1.97 ha, median = 1.14 ha). When a bee was captured, the stopwatch was paused to place the individual in a vial and to record the relevant collection information as outlined below. The effective collection time resumed once the bee was secured in the vial.

Bees were exclusively captured using nets, with the exception of honeybees (*Apismellifera*), which were not collected. Research has shown that Apidae and Megachilidae species are more frequently caught with nets than with coloured traps, whereas the reverse is true for Halictidae, which are more commonly found in traps than nets (Vandaudenard 2023). Furthermore, net collection tends to favour larger, slower species, such as bumblebees (*Bombus* spp.) (Prendergast et al. 2020). While combining net collection with pan traps may seem like a viable alternative, pan traps were not used in this study due to several limitations. When left unattended between monthly sampling sessions, traps are often stolen or overturned, either by humans or large herbivores present in the Park. Additionally, smaller specimens collected in these traps can be challenging to identify due to prolonged exposure to water, which hampers the use of identification criteria, particularly those related to the general aspect and colour of body hair. It is also important to note that net sampling provides valuable information about the plant species visited by the bees.

The captures were carried out along a variable transect, where the collector moved freely across the site, primarily guided by the presence of flowers or nesting sites, rather than following a linear transect, where the collector remained on a straight line across the site. The variable transect approach enhances the capture of bee diversity by allowing the collector to focus on key plants and areas with abundant flowering (Westphal et al. 2008). Collected individuals were placed in vials containing ethyl acetate-soaked paper for euthanisation. During collection, data such as altitude, longitude, latitude, site reference, bee behaviour (whether in flight, on the ground or on flowers) and, when applicable, the plant species visited were recorded. The sampling protocol also required specific weather conditions. Thus, all captures were conducted between 9 AM and 5 PM, with temperatures above 15°C, no wind or rain and low cloud cover.

**Specimen curation and analyses**

One day after euthanasia, the specimens were pinned dorsally through the mesosoma. For males, genitalia were extracted from the metasoma using pins, as this organ is often essential for species identification (e.g. Rasmont et al. (2021), Wood (2023)). All bees were identified to species level using identification keys under a binocular microscope and each identification was subsequently validated by a taxonomist expert of the group. Specifically, Apidae identifications were confirmed by Frédéric Carion, Guillaume Ghisbain and Achik Dorchin, Megachilidae by Clément Tourbez, Halictidae by Thomas Brau and Simone Flaminio, Andrenidae by Thomas Wood, Colletidae by Romain Le Divelec and Melittidae by Maxence Gérard.

After all specimens were identified, an accumulation curve was generated using the iNEXT package (Hsieh et al. 2016) to illustrate the rate of species discovery relative to sampling effort. This method allows for extrapolation of the effort required to detect additional species. Finally, we estimated the total number of species in the region by following the methodology proposed by Chao (1984), Chao (1987).

All records collected for the present work were published through GBIF (Gérard 2025).

## Checklists

### Checklist of the wild bees of the National Park of the Semois Valley (Belgium)

#### Andrena afzeliella

(Kirby, 1802)

03EA1796-94FC-5136-95A8-FEDCF01A2268

##### Conservation status

NE

#### Andrena angustior

(Kirby, 1802)

7954A668-7B40-5C90-AD91-0907E9A5853F

##### Conservation status

NT

#### Andrena chrysosceles

(Kirby, 1802)

A9676DE2-38DC-511B-9FB0-2746F267E717

##### Conservation status

LC

#### Andrena cineraria

(Linnaeus, 1758)

40C87643-08E4-5BFB-AD18-20E18630AAF3

##### Conservation status

LC

#### Andrena clarkella

(Kirby, 1802)

C4EFB2FD-DD2D-5158-8C56-6B18EA1DBA15

##### Conservation status

LC

#### Andrena coitana

(Kirby, 1802)

FC9CB66B-C1B3-5FE3-B40C-3ED406CDD2D8

##### Conservation status

EN

##### Distribution

This species is found in Europe, Turkey and as far east as Japan (Tomozei 2014). In Belgium, it can be found near the German border, in the southern Ardenne and in the Belgian Lorraine (Pauly 2024). To support the conservation of *Andrenacoitana*, forest management should focus on promoting habitat heterogeneity by maintaining a mix of closed and open areas (Eckerter et al. 2022). Targeted actions, such as controlled grazing and selective cutting, should be employed to slow succession and promote gradual edges where possible. Habitat creation or enhancement should be prioritised in areas with light soils, particularly those adjacent to rivers. To preserve its nesting habitats, riparian sites should be safeguarded against artificialisation.

##### Notes

The most frequently reported habitats for this species include forest edges, clearings and clear-cuts (Westrich 2018), but moorlands and coastal grasslands have also been reported (Falk and Lewington 2019). One specimen was collected in a quarry surrounded by woodlands, while the other was found in a tall-herb community within humid meadows. Nesting sites are typically solitary or found in small groups, often associated with light, well-drained soils under tree cover (Westrich 2018). Nests may sometimes be located beneath a layer of moss (Chambers 1949). Other reports indicate that the species may nest in flat riverine environments, within dry and pebbly soils (Stelfox 1927). Although this species is linked to forested environments, its pollen sources primarily come from herbaceous plants rather than trees or shrubs, with the exception of *Rubus* species (Müller 2018). Its diet is polylectic, incorporating herbs from a broad range of seven to twelve plant families, such as Apiaceae (e.g. *Angelicasylvestris*, *Daucuscarota*, *Heracleumsphondylium*), Asteraceae (e.g. *Picris*, *Cirsium*), Campanulaceae (e.g. *Campanula*, *Jasione*), Plantaginaceae (e.g. *Digitalis*) and Rosaceae (e.g. *Potentillaanserina*, *Rubus*). *Andrenacoitana* is univoltine, flying from June to August (Peeters 2012).

##### Diagnosis

**Figure 3.**
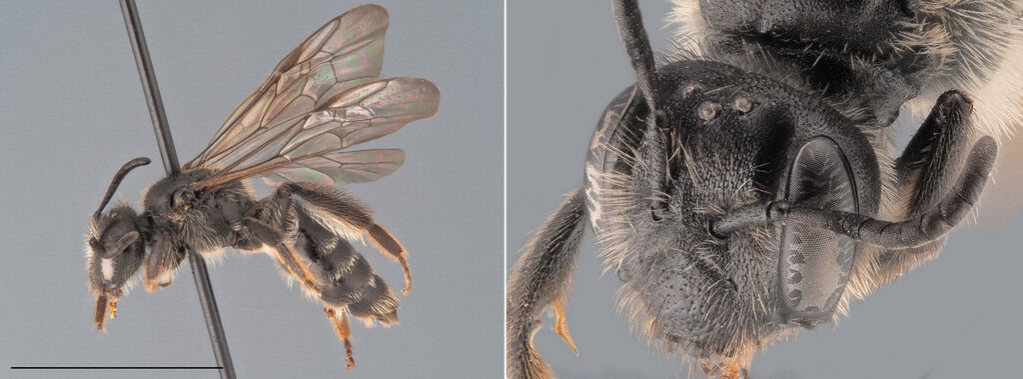
*Andrenacoitana*, ♀. Habitus in lateral view and head in oblique view. Scale bar: 5 mm. Photo credit: Paolo Rosa.

One of the small (< 9 mm) dark *Andrena* that does not belong to the *Micrandrena* subgenus (Fig. 3). Females can be identified thanks to their deep foveae, which extend up to the hind margin of the lateral ocelli. The metasoma features bands of white hairs which are interrupted medially. The propodeum is not delimited by carinae laterally. The metabasitarsi and tibiae are black. Males have a pale clypeus and pale lower para-ocular areas. Their genae are relatively short, being as wide as the compound eyes. Their third antennal flagellomere is as long or shorter than the subsequent two. The first tergite is not strongly shagreened (Wood 2023).

#### Andrena denticulata

(Kirby, 1802)

91B7DD45-3C79-553B-9253-3A5485C81D5D

##### Conservation status

NT

#### Andrena dorsata

(Kirby, 1802)

D39A9612-9037-504C-B295-7FEEBE296F65

##### Conservation status

LC

#### Andrena flavipes

Panzer, 1799

5C4A88DE-9AA2-5228-B49D-BC6568FBA811

##### Conservation status

LC

#### Andrena fucata

Smith, 1847

ED0D4598-8E9B-5D49-AD9C-BEBD91B0C4FE

##### Conservation status

VU

#### Andrena fulvago

(Christ, 1791)

C8DCF86F-7B6D-5F7C-BD1A-B9EED2854348

##### Conservation status

NT

#### Andrena gravida

Imhoff, 1832

E18C75B1-D070-5301-95CE-887EBD307873

##### Conservation status

LC

#### Andrena haemorrhoa

(Fabricius, 1781)

770E490D-95FE-5B5C-A0F7-4EC68656921C

##### Conservation status

LC

#### Andrena helvola

(Linnaeus, 1758)

CCC54107-9153-55A4-9DA0-42DED21F2A52

##### Conservation status

VU

#### Andrena humilis

Imhoff, 1832

643604F0-267B-59D4-B31A-42602C865AB3

##### Conservation status

LC

#### Andrena labialis

(Kirby, 1802)

664300E7-1296-57C4-A38B-EC259E2010B1

##### Conservation status

NT

#### Andrena lathyri

Alfken, 1899

65C8C547-43B6-5E0A-8DC7-D7B032B4FB41

##### Conservation status

NT

#### Andrena minutula

(Kirby, 1802)

7D94C912-31A5-5F39-9A21-C8145AE0B4ED

##### Conservation status

LC

#### Andrena nigroaenea

(Kirby, 1802)

085BF648-E800-5BB7-89A1-E3E5B674251F

##### Conservation status

LC

#### Andrena nitida

(Müller, 1776)

69B9E48C-70F7-5061-910C-C4B363B57AEB

##### Conservation status

LC

#### Andrena ovatula

(Kirby, 1802)

7B9CDC87-F831-5703-A06B-0AF56D90BAFE

##### Conservation status

NT

#### Andrena rosae

Panzer, 1801

DD4F7BB6-39F8-5848-A8F8-802CF4BEED25

##### Conservation status

LC

#### Andrena schencki

Morawitz, 1866

0BC3DF53-9CCB-55B8-8CEC-7EF193DFCA61

##### Conservation status

EN

##### Distribution

This species is found in Europe, in the Middle East and as far east as Turkmenistan (Tomozei 2014). *Andrenaschencki* was widespread in Belgium during the first half of the last century, but is now limited to the Ardenne and the Belgian Lorraine (Pauly 2024). Given its preference for oligotrophic grasslands, this species may benefit from the restoration of extensive agricultural practices and the reduction of nitrogen inputs.

##### Notes

This species is described as associated with oligotrophic grasslands (Westrich 2018). The only specimen we collected was found in a mesic grassland with an intermediate nutrient concentration. This observation suggests the species may occupy a broader ecological niche and that extensively managed grasslands, even with moderately elevated nutrient levels, can support its habitat requirements. Nests are typically excavated in compact, sparsely vegetated soils, such as pathways, although they can occasionally be found in lighter soils. Nests may be solitary or form aggregations of up to 100 individuals (Westrich 2018). Females are polylectic, foraging on flowers from at least five plant families: Asteraceae, Cornaceae, Brassicaceae, Dipsacaceae, and showing a preference for Fabaceae (Radchenko 2015). *Andrenaschencki* is univoltine, flying from April to July (Peeters 2012).

##### Diagnosis

**Figure 4.**
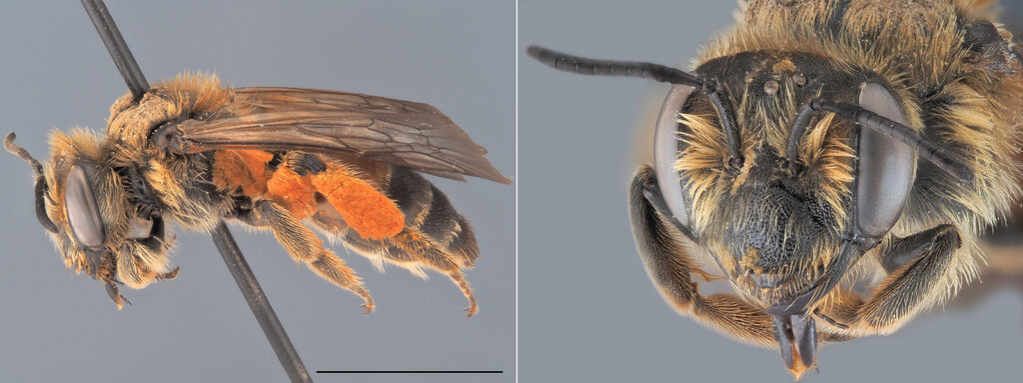
*Andrenaschencki*, ♀. Habitus in lateral view and head in oblique view. Scale bar: 5 mm. Photo credit: Paolo Rosa.

*Andrenaschencki* is one of the few Belgian *Andrena* characterised by a red-marked metasoma (Fig. 4). Both sexes are relatively large, measuring over 10 mm. Females lack plumose hairs on their hind tibiae and possess a shiny, strongly punctuated and short-haired second tergite, along with a dull and densely punctate clypeus. Males have a pale clypeus and pale para-ocular areas, as well as elongated mandibles that cross apically when closed (Wood 2023).

#### Andrena scotica

Perkins, 1916

F595FA3B-3CEF-53BD-A5EC-4BA57C26E17D

##### Conservation status

LC

#### Andrena subopaca

Nylander, 1848

6861828E-2225-5679-ADE7-2B435BCBB257

##### Conservation status

LC

#### Andrena tarsata

Nylander, 1848

2692F146-D819-5AA9-94A8-8E842F93FD20

##### Conservation status

EN

##### Distribution

This species is found across most of Europe, and as far east as China (Tomozei 2014). In Belgium, it could be observed in most of the Ardenne before 1950. Since then, observations have been extremely scarce (Pauly 2024). This species is likely threatened by habitat destruction and degradation, although its specific requirements are not fully understood. Efforts should be made to identify and protect aggregations in potential habitats such as sandy heathlands and moors. These focal habitats could also be extended or restored where possible.

##### Notes

This species has been reported in meadows, heathlands, and moors situated on sandy soils, where nests are likely to form aggregations, although few observations have been documented (Else 2005, Westrich 2018). Nests are preferentially located on sloped, south-facing surfaces (Else 2005), similar to the quarry where we collected the sole specimen of this species. While Westrich 2018 notes that females collect pollen exclusively from *Potentilla* flowers, data from England suggest that the species might visit a wider range of species (Else 2005). Adults are typically observed from June to September (Peeters 2012).

##### Diagnosis

**Figure 5.**
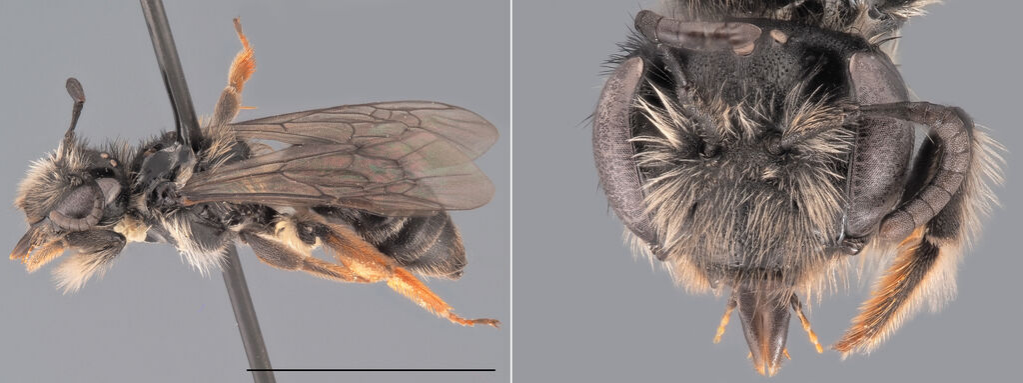
*Andrenatarsata*, ♀. Habitus in lateral view and head in frontal view. Scale bar: 5 mm. Photo credit: Paolo Rosa

Another small (< 9 mm), dark *Andrena* not belonging to the *Micrandrena* subgenus (Fig. 5). Females can be identified thanks to their orange hind tibiae and tarsi which is unique in Belgium for such a small species. Males have a pale clypeus, a short head and dark hairs along the inner eye margin. Their third antennal flagellomere is longer than the subsequent two (Wood 2023).

#### Andrena vaga

Panzer, 1799

E81F75FD-016F-53C5-B2F3-D5A0CBBE7A79

##### Conservation status

LC

#### Andrena wilkella

(Kirby, 1802)

DDFFCB0A-D49B-5475-83D2-4582BCD50980

##### Conservation status

NT

#### Panurgus banksianus

(Kirby, 1802)

E74659F7-DE79-5040-95F7-71795FCEE61D

##### Conservation status

LC

#### Panurgus calcaratus

(Scopoli, 1763)

771E79F4-D8AB-54A3-9419-F81A5F6284D5

##### Conservation status

LC

#### Anthophora furcata

(Panzer, 1798)

B0432614-B090-562E-97EF-6040DB82D5A9

##### Conservation status

LC

#### Anthophora plumipes

(Pallas, 1772)

25652E32-99A1-5E42-8F1A-17F8C74804DA

##### Conservation status

LC

#### Bombus bohemicus

Seidl, 1838

5525EE58-618A-5782-9910-0B7D99C92831

##### Conservation status

NT

#### Bombus campestris

(Panzer, 1800)

858D8278-D04A-5C59-87AE-FFC216843653

##### Conservation status

VU

#### Bombus hortorum

(Linnaeus, 1761)

4040D04E-57F9-5E45-BA4B-9BF2F5E8EE79

##### Conservation status

NT

#### Bombus humilis

Illiger, 1806

BAE29EA7-6873-5375-8040-9A88D519313E

##### Conservation status

CR

##### Distribution

*Bombushumilis* has a large Palearctic distribution (Rasmont et al. 2021). In Belgium, however, the species has vanished from nearly all the localities where it was found a hundred years ago (Folschweiller 2020). The subsisting locations are largely concentrated in the south-easternmost part of the country, in the Belgian Lorraine, with a few scarce observations east of the Sambre and Meuse valley. Habitat degradation is considered as the most threatening factor for the species, but climate change is also expected to induce substantial reductions of its European range by the end of the century (Rasmont et al. 2015, Ghisbain et al. 2024).

##### Notes

The species is mostly associated with open flower-rich grasslands and mostly collects pollen on plants from the Fabaceae, Lamiaceae and Boraginaceae families (Rasmont 1988, Folschweiller 2020, Rasmont et al. 2021, Wood et al. 2021). We collected a single individual, a queen, foraging on tufted vetch (*Viciacracca*, Fabaceae) within mesic grasslands. Folschweiller (2020) cite *Bombushumilis* as frequently in sympatry with *B.sylvarum* and *B.veteranus*, two strongly-threatened species at the Belgian scale.

##### Diagnosis

**Figure 6.**
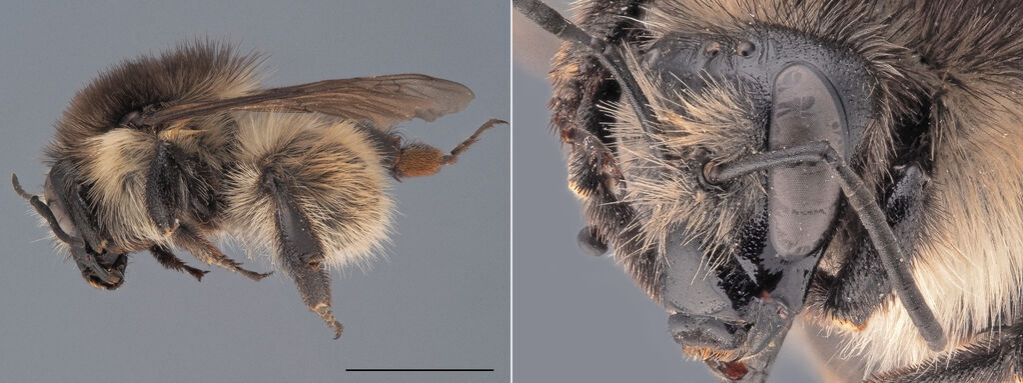
*Bombushumilis*, ♀. Habitus in lateral view and head in oblique view. Scale bar: 5 mm. Photo credit: Paolo Rosa.

*Bombushumilis* is amongst the most polymorphic bumblebee species of Europe (Fig. 6). The females of the species can be mostly confused with local colour forms of other bumblebee species belonging to the subgenus Thoracobombus, especially *B.pascuorum* and *B.muscorum*. In the females of *B.humilis*, however, the area directly in front of the central ocellus is largely smooth, with only a few punctuations, while this area is much more punctured in *B.pascuorum* and *B.muscorum*. In addition, the hairs on tergite 6 of *B.humilis* females are black, thick and erect, while they are ginger, thin and more parallel to the cuticle in *B.pascuorum*. The males of *B.humilis* can be differentiated from the males of all other bumblebee species on the basis of the morphology of their genitalia (drawn in Rasmont et al. 2021).

#### Bombus hypnorum

(Linnaeus, 1758)

38A09FCF-EC4D-5CC2-B89B-084711ECDE5F

##### Conservation status

LC

#### Bombus lapidarius

(Linnaeus, 1758)

CB0EF477-9827-5A53-A6C5-1599815207E1

##### Conservation status

LC

#### Bombus lucorum

(Linnaeus, 1761)

19F61EAD-D0F2-538A-9C11-BB83256139E4

##### Conservation status

NT

#### Bombus norvegicus

Schneider, 1918

F5CAC81D-E881-52E0-B7A8-50A9FD9A0981

##### Conservation status

VU

#### Bombus pascuorum

Scopoli, 1763

17711327-973A-5C42-A7E4-C2531FFA5CE0

##### Conservation status

LC

#### Bombus pratorum

(Linnaeus, 1761)

C0036EB2-58BE-5D54-A16E-9C342B34D116

##### Conservation status

LC

#### Bombus ruderarius

(Müller, 1776)

4ED3C3E7-6A0F-585E-872C-EADB53B37EA2

##### Conservation status

EN

##### Distribution

*Bombusruderarius* has a wide distribution in the West Palearctic (Rasmont et al. 2021). In Belgium, the species has been strongly regressing over the last century (Folschweiller 2020) although its status is less critical than that of *B.humilis*. The subsisting locations are largely concentrated east of the Sambre and Meuse valley and in the coastal area of north-western Flanders. Habitat degradation is considered as the most threatening factor for the species, but climate change is also expected to induce substantial reductions of its European range by the end of the 21^st^ century (Rasmont et al. 2015, Ghisbain et al. 2024).

##### Notes

*Bombusruderarius*, as many other species of the genus *Thoracobombus*, is mostly associated with open flower-rich grasslands. Its habitats of predilection also include coastal dunes, heaths and moors, woodland edge and clearings in woodlands. The diet of the species includes a large quantity of pollen from the Fabaceae family (Folschweiller 2020, Rasmont et al. 2021, Wood et al. 2021). Of the three individuals collected, two were foraging on plants from the Fabaceae family, and one on a plant from the Asteraceae family, all from mesic grasslands.

##### Diagnosis

**Figure 7.**
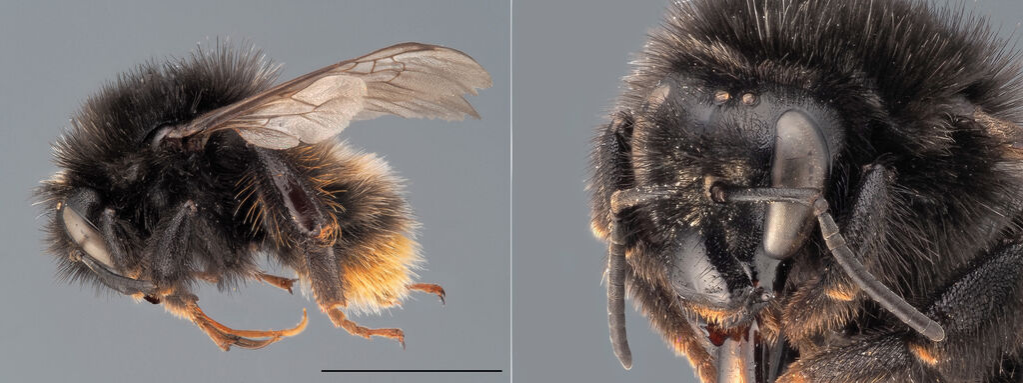
*Bombusruderarius*, ♀. Habitus in lateral view and head in oblique view. Scale bar: 5 mm. Photo credit: Paolo Rosa.

In Belgium, the females of *B.ruderarius* (Fig. 7) can be confused with other species of black bumblebees with a ‘red tail’, such as *B.lapidarius*, *B.rupestris*, *B.soroeensis* and *B.cullumanus* (although the latter is thought to be fully extinct in the country). However, females of *B.ruderarius* can be separated from all other concolour bumblebees by the combined presence of: (i) the mid-basitarsus with the distal posterior corner extended to form a sharp angle and (ii) orange fringes of hairs on the corbicula. The males of *B.ruderarius* can be differentiated from the males of all other bumblebee species on the basis of the morphology of their genitalia (drawn in Rasmont et al. 2021).

#### Bombus rupestris

Fabricius, 1793

62A8841E-249F-5B48-BADA-5ACBE9E3C526

##### Conservation status

EN

##### Distribution

*Bombusrupestris* has a large distribution in the West Palearctic (Rasmont et al. 2021). In Belgium, the species is absent from many localities where it was once found (Folschweiller 2020), especially in the Hainaut province. Most of its subsisting locations are concentrated in the northern and oriental parts of its Belgian distribution (Campine, Ardenne and Belgian Lorraine). It is noteworthy that, even historically, *B.rupestris* has never been a relatively abundant species in the country. The males of the species are largely associated with thistles and their regression could have acted as a major factor of decline in the species. Besides, climate change is expected to induce large-scale reductions of its European range by 2080-2100 (Rasmont et al. 2015, Ghisbain et al. 2024).

##### Notes

*Bombusrupestris* is a socially parasitic bumblebee species. It parasitiszes the nest of *Bombuslapidarius*, a very common species both across Europe and in Belgium (Rasmont et al. 2021). The species does not seem to be strongly associated with specific habitat types, although it is largely absent from urbanised areas (Folschweiller 2020).

##### Diagnosis

**Figure 8.**
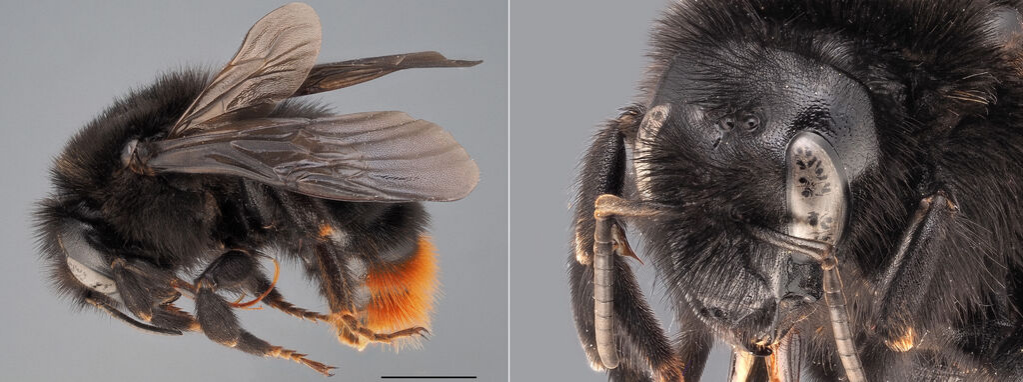
*Bombusrupestris*, ♀. Habitus in lateral view and head in oblique view. Scale bar: 5 mm. Photo credit: Paolo Rosa.

In Belgium, the females of *B.rupestris* (Fig. 8) can be confused with other species of black bumblebees with a ‘red tail’, such as *B.lapidarius*, *B.ruderarius*, *B.soroeensis* and *B.cullumanus* (although the latter is thought to have fully extinct from the country). However, females of *B.rupestris* can be separated from all other concolour bumblebees by the presence of a high density of hairs on the metatibia. This feature is typical of the parasitic bumblebees of the subgenus Psithyrus, that lack a corbicula for collecting pollen. The males of *B.rupestris* can be differentiated from the males of all other bumblebee species on the basis of the morphology of their genitalia (drawn in Rasmont et al. 2021).

#### Bombus spp. sensu stricto

NA

589E1799-54C5-573D-8400-6CEA71F2CA63

##### Conservation status

NA

#### Bombus soroeensis

(Fabricius, 1777)

DD6FB65A-C49E-5CE6-9519-92798E1100C8

##### Conservation status

VU

#### Bombus sylvarum

(Linnaeus, 1761)

8D20B1C7-5BD9-5CF9-8BA6-FBA39CA04FCE

##### Conservation status

CR

##### Distribution

*Bombussylvarum* has a large distribution in the West Palearctic (Rasmont et al. 2021). In Belgium, the species was extirpated from many localities where it used to thrive, especially in the western part of its distribution. Most of its subsisting locations are now concentrated east of the Sambre and Meuse valley. It can be noted that, even historically, *B.sylvarum* never seemed abundant in the country (Folschweiller 2020). Although habitat degradation has likely led the Belgian populations to their current status, climate change is expected to further impact the European range of *B.sylvarum* by the end of the century (Rasmont et al. 2015, Ghisbain et al. 2024).

##### Notes

*Bombussylvarum* lives in a range of more or less open, flower-rich habitats where it can collect pollen from plants of the Fabaceae and Lamiaceae families (Rasmont et al. 2021, Wood et al. 2021). Notably, of the five individuals collected, only one was foraging on a plant from the Fabaceae family. Two were foraging on Asteraceae, one on Onagraceae, and one on Plantaginaceae. Folschweiller 2020 cite the species as frequently in sympatry with *B.humilis* and *B.veteranus*, two strongly-threatened species at the Belgian scale.

##### Diagnosis

**Figure 9.**
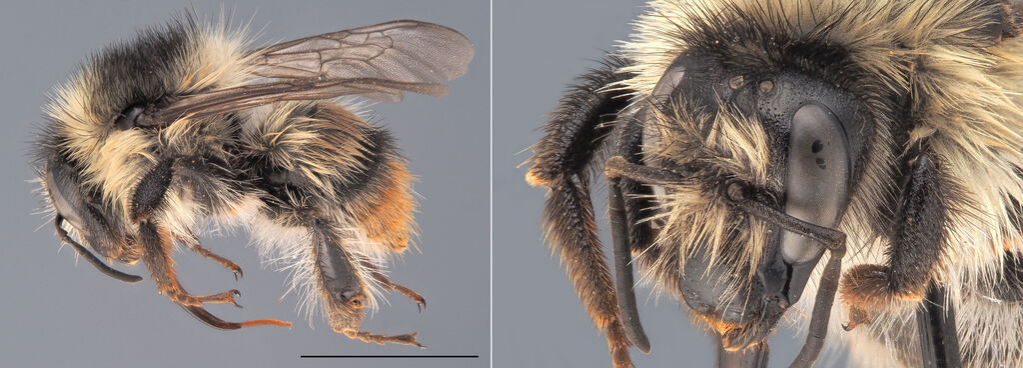
*Bombussylvarum*, ♀. Habitus in lateral view and head in oblique view. Scale bar: 5 mm. Photo credit: Paolo Rosa.

In Belgium, the females of *Bombussylvarum* (Fig. 9) can only be confused with those of *B.veteranus*. In the latter, the basis of the tergites 3 to 5 always include black hairs, which is not the case in the females of *B.sylvarum*. Furthermore, the mandibles of *B.veteranus* are substantially elongated, while those of *B.sylvarum* have a regular size, comparable to those of other females of free-living bumblebees. The males of *B.sylvarum* can be differentiated from the males of all other bumblebee species on the basis of the morphology of their genitalia (drawn in Rasmont et al. 2021).

#### Bombus sylvestris

(Lepeletier, 1833)

4202282D-410A-573E-B7C1-BDC32B412AE8

##### Conservation status

LC

#### Bombus vestalis

(Geoffroy in Fourcroy, 1785)

B7A3751A-54C2-5887-91B5-89065B8EE49F

##### Conservation status

NT

#### Ceratina cyanea

(Kirby, 1802)

2F84F13F-527F-5A35-831D-D0ACC6919BED

##### Conservation status

LC

#### Epeoloides coecutiens

(Fabricius, 1775)

E1D13FF9-47A3-51C9-90A7-F1744DC40221

##### Conservation status

LC

#### Eucera longicornis

(Linnaeus, 1758)

230D5599-A1EE-5056-B02B-742A519D6F33

##### Conservation status

VU

#### Eucera nigrescens

Pérez, 1880

AFA3C677-D900-55B1-AE4E-F4D762EB63C5

##### Conservation status

EN

##### Distribution

Its population in Belgium has declined around 50% to 80% between 1900-1969 and 1970-2017, with severely fragmented populations (Drossart 2019). Few populations remain in Pays de Herve, Condroz and Plateaux Limoneux Hennuyers (Drossart 2019). The drastic decline of leguminous crops and nutrient-poor grasslands is probably a major driver of this decline.

##### Notes

*Euceranigrescens* primarily inhabits meadows and grasslands rich in Fabaceae, as these plants are its main foraging source (Standfuss 2009). Females exclusively collect pollen from Fabaceae species, while males are often associated with the orchid *Ophrysfuciflora* (Gaskett 2010). The single specimen we collected was observed foraging on a *Lathyrus* species.

##### Diagnosis

**Figure 10.**
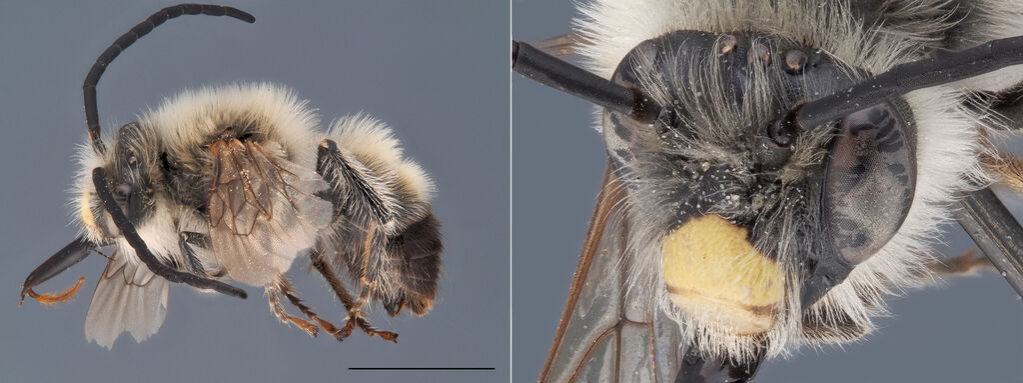
*Euceranigrescens*, ♂. Habitus in lateral view and head in oblique view. Scale bar: 5 mm. Photo credit: Paolo Rosa.

One of the two *Eucera* species in Belgium. Compared to *E.longicornis*, *E.nigrescens* (Fig. 10) it has a domed and elongated clypeus, and the mesonotum weakly punctuated (*Amiet et al. 2007*). In males, the mesosoma hair colour varies considerably, ranging from brown to silver, whereas, in females, the variation is more limited, primarily spanning from light to dark brown.

#### Nomada braunsiana

Schmiedeknecht, 1882

93B9E9FB-2423-5744-A44E-6C4B4AD25A9D

##### Conservation status

NE

#### Nomada flava

Panzer, 1797

100E0D1B-B1E9-5AC5-8EF3-DC294FBE2DEC

##### Conservation status

LC

#### Nomada flavoguttata

(Kirby, 1802)

BB4A7993-E4B3-501E-988B-7DA67130806F

##### Conservation status

LC

#### Nomada fucata

Panzer, 1798

B3339F1C-AC0F-5B36-A4D1-3597933B150D

##### Conservation status

LC

#### Nomada fulvicornis

Fabricius, 1793

DDCEBF54-EA3D-574C-AA24-A586E4638C81

##### Conservation status

LC

#### Nomada goodeniana

(Kirby, 1802)

07C77877-D47A-59DA-A8CB-4250C0D65463

##### Conservation status

LC

#### Nomada leucophthalma

(Kirby, 1802)

CC128BD8-870C-5F8B-B4E8-241017CCC850

##### Conservation status

LC

#### Nomada ruficornis

(Linnaeus, 1758)

D06D7011-B260-5B14-B80F-59284612FB6B

##### Conservation status

LC

#### Nomada rufipes

Fabricius, 1793

DA78EFA3-C8CA-5A1A-942E-36C5FB5ABC00

##### Conservation status

NT

#### Nomada sexfasciata

Panzer, 1799

65F37CD4-15B5-5210-9C5F-9AB3141A0825

##### Conservation status

CR

##### Distribution

Recent records of *N.sexfasciata* in Belgium are largely restricted to the Fagne-Famenne geological region and the south-eastern part of the Campine. Its populations have declined by more than 80% between 1900–1969 and 1970–2017, particularly around Brussels and in the Province of Liège (Drossart 2019). However, this species has always been rare and habitat degradation - especially the loss of Fabaceae-rich grasslands, which support its host species - is likely the main threat to its continued survival.

##### Notes

*Nomadasexfasciata* is a brood parasite of two threatened species in Belgium, both sampled during our inventory: *Euceralongicornis* and *E.nigrescens* (Westrich 2008). It shares the same habitats as its hosts, primarily grasslands rich in Fabaceae species.

##### Diagnosis

**Figure 11.**
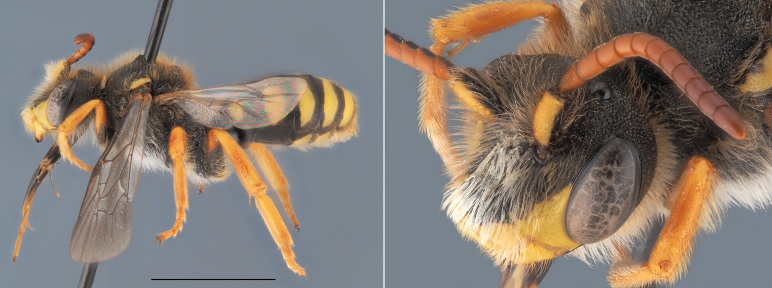
*Nomadasexfasciata*, ♀. Habitus in lateral view and head in oblique view. Scale bar: 5 mm. Photo credit: Paolo Rosa.

This species of *Nomada* is relatively large, measuring between 12 and 14 mm in length (Fig. 11). The metasoma is entirely black, with prominent yellow lateral patches on the first three tergites. The mesosoma is notably hairy for a *Nomada* species and bears two yellow spots on the scutellum. A distinctive characteristic of this species, compared to other species from Belgium, is its particularly inflated clypeus (seen from the side), and relatively wide malar gap between the eyes and the base of the mandible (Smit 2018, Falk and Lewington 2019).

#### Nomada signata

Jurine, 1807

87C1E595-4B4E-5AB5-8D55-814709E65F6E

##### Conservation status

LC

#### Nomada succincta

Panzer, 1798

FA1EB1D6-E1C9-50F1-A997-7E62628E9738

##### Conservation status

LC

#### Colletes cunicularius

(Linnaeus, 1761)

B1FC5E31-B8A8-5C95-950B-CD3B09616998

##### Conservation status

LC

#### Colletes daviesanus

Smith, 1846

BB08BB5F-4184-583A-AA6A-265B8AE3A46A

##### Conservation status

LC

#### Hylaeus communis

Nylander, 1852

84E08412-52BA-5B56-AA11-801F7E6AD554

##### Conservation status

LC

#### Hylaeus confusus

Nylander, 1852

29F25CE6-BD8A-54B3-AC1B-265B664D2B78

##### Conservation status

LC

#### Hylaeus difformis

(Eversmann, 1852)

4F773D76-D769-538E-8BAF-3438478B4A46

##### Conservation status

LC

#### Hylaeus incongruus

Forster, 1871

CD8274F2-9D7E-5AE5-917B-C7DA7D620794

##### Conservation status

DD

#### Hylaeus rinki

(Gorski, 1852)

881B32A3-920B-5456-9CCA-1F2A00F189E0

##### Conservation status

VU

#### Halictus maculatus

Smith, 1848

A80AF63D-1EA6-58C4-B6B5-4718AD4D808B

##### Conservation status

VU

#### Halictus rubicundus

(Christ, 1791)

DBB0F4C9-84C9-5544-A70D-C2B9CE536B27

##### Conservation status

LC

#### Halictus scabiosae

(Rossi, 1790)

6A5EE3B9-7BE9-51B1-BD08-155557C776AE

##### Conservation status

LC

#### Halictus sexcinctus

(Fabricius, 1775)

47BF9243-E15E-5664-A3FD-D3256DCEB88E

##### Conservation status

VU

#### Halictus simplex

Blüthgen, 1923

BCF9034F-E17A-5DBC-91A9-0813B1ED3B95

##### Conservation status

EN

##### Distribution

This species has a distribution range extending from Spain to Kazakhstan, with occurrences decreasing in northern Europe (Pauly et al. 2016). In Belgium, *H.simplex* was recorded across most provinces of Wallonia during the last century. Recently, however, observations of this species have been largely restricted to the Ardennes (Pauly 2017). Comparative studies estimate a substantial decline in its area of occupancy, ranging from 50% to 80%, between the periods 1900–1969 and 1970–2017 (Drossart 2019). This decline is likely due to habitat destruction, highlighting the need for restoration efforts. To support *H.simplex*, restoration of thermophilic, nutrient-poor grasslands should be prioritised, with measures promoting extensive land-management practices.

##### Notes

This species inhabits calcareous grasslands and a broader range of thermophilic environments with sparse vegetation, including sand and gravel pits (Westrich 2018). Nests are constructed on horizontal surfaces and feature a short, above-ground tube structure (Pesenko et al. 2000). While three specimens were collected in quarries, the other two were found in wet grasslands crossed by a river with a gravel-based riverbed, likely providing favourable nesting sites. The species might be primitively eusocial, but this trait has to be confirmed (Pesenko et al. 2000). The female is polylectic with a preference for species within the families Asteraceae (*Cichorium* and *Centaurea*) and Dipsacaceae (*Knautia*, *Succisa*, *Scabiosa*) (Pauly and Vereecken 2018, Westrich 2018). While three of the specimens collected were indeed foraging on Asteraceae plants, one of them was sampled on *Digitalispurpurea* (Plantaginaceae). Females emerge from diapause in April, while males fly from June onwards (Westrich 2018).

##### Diagnosis

**Figure 12.**
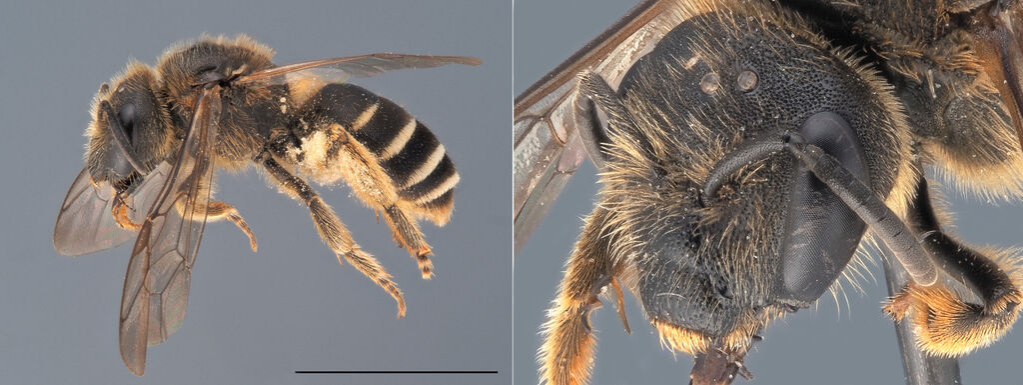
*Halictussimplex*, ♀. Habitus in lateral view and head in oblique view. Scale bar: 5 mm. Photo credit: Paolo Rosa.

A medium-sized *Halictus* (10-11 mm) with dark legs and a densely- punctuated scutum (Fig. 12). Females cannot be distinguished from the closely related *Halictuscompressus* and *H.langobardicus*, based on morphology. The lateral faces of the propodeum are conspicuously punctuated. The apical strips of hairs on tergites 1 and 2 are interrupted medially. Males feature concave genae and unmodified, thin mandibles (Pauly 2015a).

#### Lasioglossum albipes

(Fabricius, 1781)

828B67E9-01D6-52BF-A93B-97C7F2648DF6

##### Conservation status

NT

#### Lasioglossum calceatum

(Scopoli, 1763)

F235DA3C-8CC4-530A-90DE-E7D828EFC386

##### Conservation status

LC

#### Lasioglossum costulatum

(Kriechbaumer, 1873)

9C1A1C1B-B866-5D9E-9726-2714066C444B

##### Conservation status

CR

##### Distribution

*Lasioglossumcostulatum* reaches the septentrional edge of its distribution in Poland and Belgium, extending southwards to Morocco and as far east as Irkutsk (Russia). In Belgium, this species has always been very rare and is now restricted to the Gaume Region (Pauly and Vereecken 2018). Restoring this species’ habitat involves maintaining and expanding forest gaps in order to enhance the abundance and diversity of Campanulaceae (Braun‐Reichert et al. 2021) .

##### Notes

This species is mainly observed along forest edges, in thermophilic habitats such as nutrient-poor meadows, quarries and railway embankments - though one of the specimens was also collected on a wet grassland. Nests are constructed in soils rich in sand or loess (Westrich 2018). The Campanulaceae are its preferred source of pollen (Pesenko et al. 2000). However, we observed it on three other plant families, where it may have been foraging for nectar. Although *L.costulatum* is generally considered solitary, some evidence suggests it may form communal aggregations (Pesenko et al. 2000). Females emerge from diapause in late April, while males begin flying from July onwards (Westrich 2018).

##### Diagnosis

**Figure 13.**
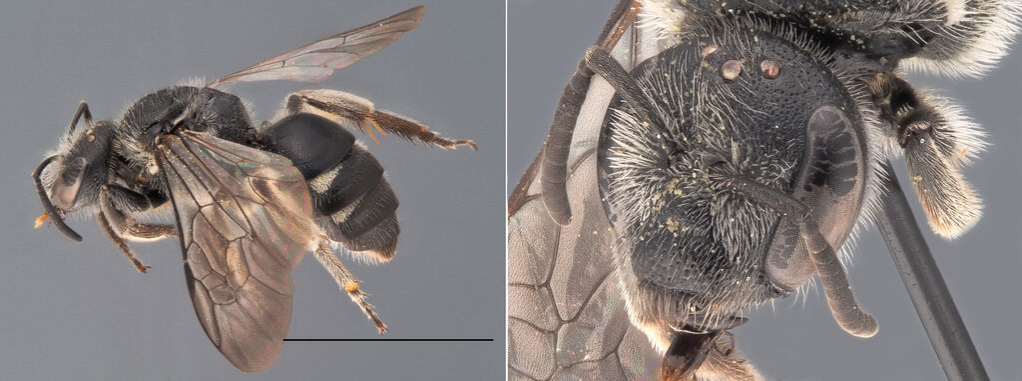
*Lasioglossumcostulatum*, ♀. Habitus in lateral view and head in oblique view. Scale bar: 5 mm. Photo credit: Paolo Rosa.

This species is one of the many *Lasioglossum* featuring tergites with dark hind margins and a carinate propodeum (Fig. 13). Both males and females can be identified by the hexagonal shape of the posterior face of their propodeum, which features strong parallel wrinkles (Pauly 2015a).

#### Lasioglossum laticeps

(Schenck, 1869)

7D61653D-E578-53FF-88F7-0EF3B4C711F6

##### Conservation status

LC

#### Lasioglossum lativentre

(Schenck, 1853)

79580667-FAC1-5B16-A7CC-A1C8B6EC070E

##### Conservation status

LC

#### Lasioglossum leucopus

(Kirby, 1802)

B9CC8AE9-030D-5F5A-974A-A783C32A64A8

##### Conservation status

NT

#### Lasioglossum leucozonium

(Schrank, 1781)

B9D464E7-26ED-5D24-A7C2-0300D0BA8B79

##### Conservation status

LC

#### Lasioglossum majus

(Nylander, 1852)

0C657819-E4C2-5ECA-83C3-0D9CBED30C80

##### Conservation status

LC

#### Lasioglossum morio

(Fabricius, 1793)

8E1CBDE1-15AC-5A5C-AF44-8393D8DA3E7E

##### Conservation status

LC

#### Lasioglossum pallens

(Brullé, 1832)

8CFD0F8F-A7AC-50FA-A9A9-075EBBA5F053

##### Conservation status

LC

#### Lasioglossum parvulum

(Schenck, 1853)

D84A1FF2-78C9-576B-AF6A-8FB226FE403C

##### Conservation status

LC

#### Lasioglossum pauxillum

(Schenck, 1853)

BC280627-9064-5775-881F-72A85981D811

##### Conservation status

LC

#### Lasioglossum punctatissimum

(Schenck, 1853)

1F30F4AE-9778-5A09-8B13-30F7D14E653B

##### Conservation status

LC

#### Lasioglossum rufitarse

(Zetterstedt, 1838)

AE98C37E-0912-5DB1-9952-275C3F1ABC41

##### Conservation status

NT

#### Lasioglossum sexnotatum

(Kirby, 1802)

5102A75F-C06C-559F-B0DD-2D0BB70864D2

##### Conservation status

LC

#### Lasioglossum villosulum

(Kirby, 1802)

E0890E32-9A3D-54BB-9C41-2F52FC382962

##### Conservation status

LC

#### Lasioglossum zonulum

(Smith, 1848)

3CACEA66-1BD1-5AF9-B42B-E6C5CCB9EE6E

##### Conservation status

LC

#### Seladonia tumulorum

(Linnaeus, 1758)

F73EC9D3-AB5F-5201-8BF6-D0E2536EFBFE

##### Conservation status

LC

#### Sphecodes crassus

Thomson, 1870

A387089A-22A8-5B23-92ED-CED780B13396

##### Conservation status

LC

#### Sphecodes ephippius

(Linnaeus, 1767)

06DBE8AC-1F69-5B4F-B72D-FE08116A12D8

##### Conservation status

LC

#### Sphecodes geoffrellus

(Kirby, 1802)

12057128-E9D2-5F5F-B831-9551C1BF9E17

##### Conservation status

LC

#### Sphecodes monilicornis

(Linnaeus, 1758)

DC1436D7-ECF8-5735-9EB4-C4AAA3C731E7

##### Conservation status

LC

#### Sphecodes puncticeps

Thomson, 1870

06FA39AF-F663-5643-8885-672D14F7BD2F

##### Conservation status

LC

#### Sphecodes reticulatus

Thomson, 1870

4AFEB8B6-275B-5DFC-8294-65631C1CF415

##### Conservation status

LC

#### Anthidiellum strigatum

(Panzer, 1802)

71AB2B33-E2B6-526E-9F20-DDF052C5AFE9

##### Conservation status

LC

#### Anthidium oblongatum

(Illiger, 1806)

4D07AD97-5C9A-5099-81C5-EB84C2A50E20

##### Conservation status

LC

#### Chelostoma florisomne

(Linnaeus, 1758)

17D0BCA1-1E5C-5EF6-BD76-D6C02977DCB3

##### Conservation status

LC

#### Heriades truncorum

(Linnaeus, 1758)

FA753547-532F-5952-B1BB-BA3C4CB9DC07

##### Conservation status

LC

#### Hoplitis adunca

(Panzer, 1798)

A7380ECB-3E29-5E94-BB8F-401673FCFAA0

##### Conservation status

LC

#### Hoplitis claviventris

(Thomson, 1872)

3E62CA14-40BE-5911-9182-DE90A395C2CA

##### Conservation status

VU

#### Megachile lapponica

Thomson, 1872

6C1E106F-E065-521C-B002-638A033BC1CE

##### Conservation status

LC

#### Megachile ligniseca

(Kirby, 1802)

8DC91870-92E2-53E9-92D9-BF876B8195F5

##### Conservation status

LC

#### Megachile versicolor

Smith, 1844

87A90806-578D-5212-A088-990BB689DA4C

##### Conservation status

LC

#### Megachile willughbiella

(Kirby, 1802)

A23A184C-7AF9-5B28-917A-ED7DDA97B797

##### Conservation status

LC

#### Osmia bicolor

(Schrank, 1781)

E4A83F52-BB2A-50DA-8503-22492FED9A69

##### Conservation status

LC

#### Osmia bicornis

(Linnaeus, 1758)

70B9CE62-928F-58B5-8F41-0597B552181C

##### Conservation status

LC

#### Osmia leaiana

(Kirby, 1802)

1CC03562-5B5A-500C-822B-01F9B456C45A

##### Conservation status

LC

#### Osmia parietina

Curtis, 1828

7654A6D6-E384-52BF-BCAD-CDE9C8D56BFD

##### Conservation status

EN

##### Distribution

The population trend of *O.parietina* in Belgium is not well studied. Although Rasmont (1993) assessed its population as stable, more recent estimates suggest that the species is now declining in Belgium (Drossart 2019). Forestry practices that promote the retention of deadwood could benefit this species by providing suitable nesting habitats.

##### Notes

This species favours mixed habitats, including open, sunny areas and woodlands for nesting (Westrich 2008). Data from Belgium suggest that *O.parietina* is a generalist species, with a particular preference for Fabaceae (Westrich 2008, Pauly, pers. comm. 2015).

##### Diagnosis

**Figure 14.**
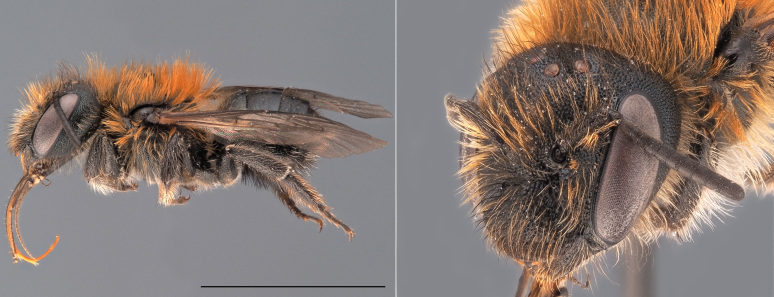
*Osmiaparietina*, ♀. Habitus in lateral view and head in oblique view. Scale bar: 5 mm. Photo credit: Paolo Rosa.

*Osmiaparietina* is a small species of Megachilidae, characterised by predominantly brown hairs on the mesosoma, interspersed with some black hairs (Fig. 14). It closely resembles *O.uncinata*, though *O.parietina* is smaller and has a less densely hairy metasoma (Pauly 2015b).

#### Trachusa byssina

(Panzer, 1798)

030A89ED-C5FE-505F-AABB-EA035739D762

##### Conservation status

LC

#### Macropis europaea

Warncke, 1973

EC557D7A-3099-5824-B22B-C71AB07D9B22

##### Conservation status

LC

#### Macropis fulvipes

(Fabricius, 1804)

74159398-D8CE-57F0-86DB-C58CC62FC7B1

##### Conservation status

LC

#### Melitta haemorrhoidalis

(Fabricius, 1775)

EE163E8B-171D-5A87-B976-9B0D68157505

##### Conservation status

LC

#### Melitta nigricans

Alfken, 1905

4C25E18A-4F88-5725-98FC-BD4E42A4271D

##### Conservation status

LC

## Analysis

**Diversity and abundance of species**

**Figure 15.**
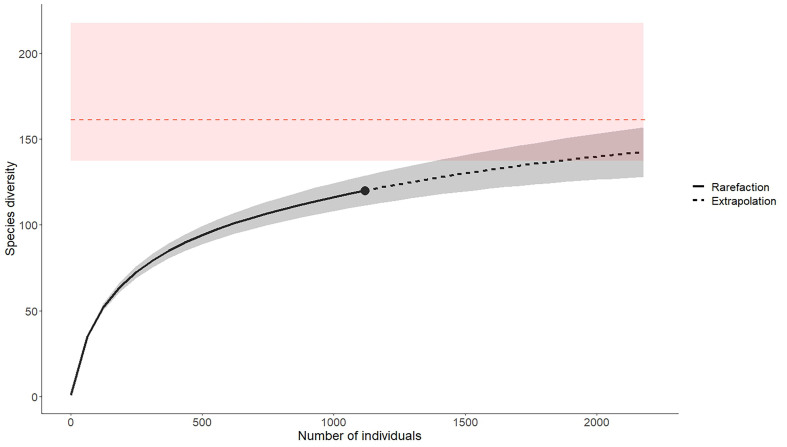
Accumulation curve and expected number of species collected in National Park of the Semois Valley (Belgium). The sampling effort is represented by the number of specimens collected (x axis). The dotted line represents the predicted number of species (y axis), based on the number of specimens collected. The total predicted species richness and the associated confidence interval (in red) are calculated using the Chao method.

Our standardised survey resulted in the collection of 1,119 specimens, representing 120 of the 419 bee species recorded in Belgium, i.e. 28.6% of the national bee fauna. By comparison, over a geographical area with a similar size, three years of sampling in the industrial region of the Hainaut Province yielded 9,410 specimens, encompassing 180 species (Fiordaliso et al. 2022). This discrepancy suggests that many species are yet to be discovered in the Semois National Park, as indicated by our accumulation curve (Fig. 15). Indeed, using the method proposed by Chao (1984, 1987), we estimate the maximum number of species in the province to be 161, with a confidence interval between 137 and 217 species (Fig. 15). Consequently, a single year of sampling has likely led to the identification of 55-88% of the species present in the National Park. The large standard error highlights that more sampling effort is needed to refine this estimation. As the species accumulation curve did not reach a plateau, direct comparisons of observed diversity with other protected areas remain challenging. However, when compared to a bee survey conducted in a European national park with similar forest-dominated habitats and a comparable position on the accumulation curve, the results appear relatively similar. For instance, in Wielkopolska National Park (Poland), 110 bee species were recorded over a six-month sampling period (Banaszak-Cibicka et al. 2018). It is important to note that Wielkopolska National Park is approximately half the size of SVNP, suggesting again that additional sampling efforts in SVNP would be necessary to enable robust conclusions.

The genus *Bombus* (family Apidae) was the most abundant in our study, with more than one-third of the individuals collected (38%, n = 424) belonging to this genus. The two most frequently collected taxa were *Bombuspascuorum* (14.92%, n = 167) and species belonging to the subgenus Bombus *sensu stricto* (5.72%, n = 64), a subgenus represented by species in which the females of some species can only be reliably distinguished through genetic or semio-chemical analyses (Rasmont et al. 2021). The third most prevalent species was another bumblebee, *B.hortorum* (5.09%, n = 57). Although widespread and abundant across Europe (Ghisbain et al. 2021b), this species is classified as Near Threatened in Belgium (NT), with populations having recently declined (Drossart 2019). Its abundance in the National Park is likely due to the well-preserved grasslands, which host a rich diversity of Fabaceae plants. Moreover, this is one of the few bumblebee species partially associated with woodland habitats (Folschweiller 2020). While the prominence of some bumblebee species suggests that a restricted set of certain common species within this group are resilient to global environmental changes (Rasmont et al. 2015), it may also be an artefact of our sampling method, as the use of nets tends to overestimate the abundance of larger, social species (Prendergast et al. 2020). Amongst the solitary bees, *Trachusabyssina* (4.2%, n = 47, Megachilidae) was the most abundant. This finding is particularly noteworthy, as the species is protected in Belgium and is rarely recorded in other studies (e.g. Fiordaliso et al. (2022), who found only five specimens amongst 9,410 bees collected). *Trachusabyssina* is a summer species largely associated with Fabaceae plants and it was primarily collected from *Lotus*, which was present in all the habitat types sampled. Additionally, *Trachusabyssina* constructs its nests in the ground using tree leaves, which it seals with conifer resin. It is, thus, one of the few solitary bee species that benefit from mixed and coniferous forests. The second most abundant solitary species was *Lasioglossumcalceatum* (3.57%, n = 40, Halictidae), one of the most common spring species in Belgium, primarily visiting *Taraxacum* spp. *Colletesdaviesanus* (2.86%, n = 32, Andrenidae), an oligolectic species on the Asteraceae family, with a summer phenology, was the third most common solitary bee in our sampling. This species is widespread and abundant in Belgium.

Of the 120 bee species collected in this survey, 25 (around 21%) are either brood parasites (e.g. *Nomada*, *Sphecodes*) or inquilines (bumblebees of the subgenus Psithyrus). Parasitic taxa constitute a diverse guild of wild bees (Michener 2007) with a great potential as indicator taxa for assessing the health of bee communities (Sheffield et al. 2013). Due to their higher trophic level, their presence depends on the presence of their hosts and the resources used by the latter. Based on Sheffield et al. 2013, a proportion exceeding 20% of parasitic taxa amongst the total bee species sampled is considered high. Further detecting the presence of these parasites, that are most generally undersampled, overlooked and understudied due to their relatively lower abundance (Ghisbain 2023), will, therefore, be critical for properly characterising the communities of the National Park.

The overall sampling includes 34 singletons, representing 28.3% of the species, a proportion notably higher than those reported for wild bee communities in literature (e.g.Williams et al. (2001), Fiordaliso et al. (2022)). This elevated number of species collected as single specimens supports the findings of the species accumulation curve, suggesting that additional sampling effort is necessary to fully capture the bee diversity of the SVNP. Additionally, some species may naturally occur at low population densities. The SVNP offers a wide range of heterogeneous microhabitats, some of which are highly fragmented among forest patches, potentially limiting the abundance of certain species by restricting access to essential resources. Furthermore, since some species have very short activity periods and each site was sampled only once per month, the low probability of catching them might explain why they are not represented in our sampling.

**Threatened and protected species**

**Figure 16.**
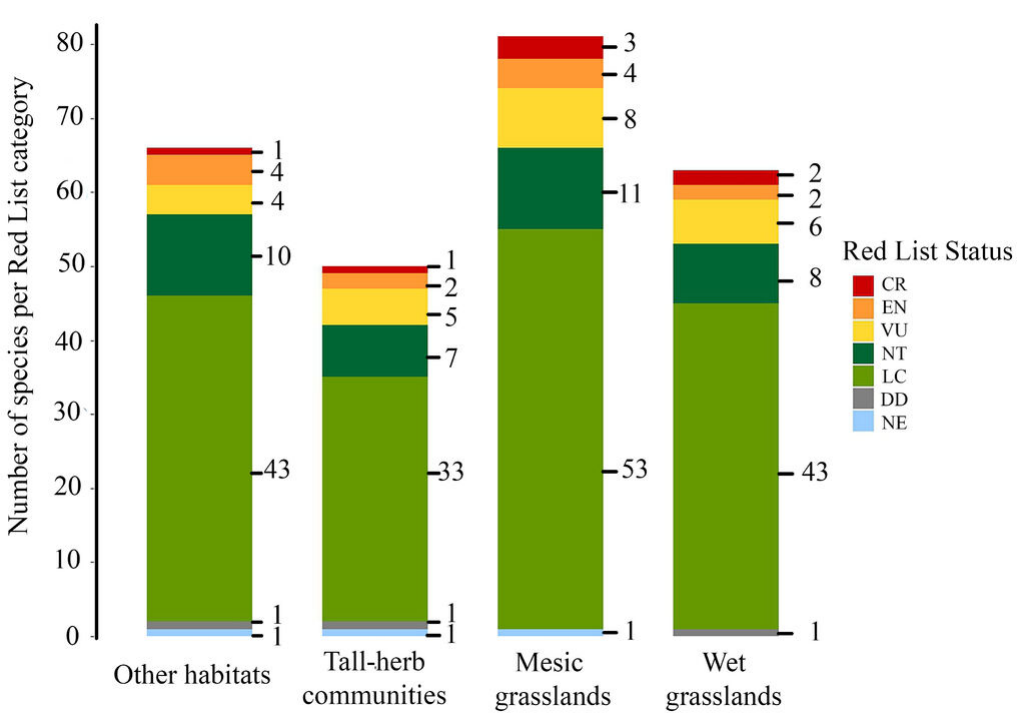
Species distribution within the four types of habitats, according to their Red List status at the Belgian scale, following Drossart (2019). CR: Critically Endangered, EN: Endangered, VU: Vulnerable, NT: Near Threatened, LC: Least Concern, DD: Data Deficient, NE: Not Evaluated.

Of the 120 species identified, 22 are classified as threatened at the national level, based on the Red List of Belgian bees (CR: Critically Endangered (n = 4), EN: Endangered (n = 8), VU: Vulnerable (n = 10); Drossart (2019)), accounting for 18.3% of the total number of species collected (Fig. 16). Amongst the most remarkable species, *Bombushumilis*, *Bombussylvarum*, *Lasioglossumcostulatum* and *Nomadasexfasciata* were the four species Red-listed as CR. All species collected and their Red List status in Belgium are listed in Table 1.

We recorded 12 species legally protected in Wallonia within the National Park. The most frequently encountered were *Trachusabyssina* (n = 47) and *Macropiseuropaea* (n = 15). The diet of *Macropiseuropaea* is highly specialised, as it forages exclusively on *Lysimachia* species - plants that thrive in particularly wet habitats - to collect oil. Its phenology is largely confined to the summer months.

Eighty-one bee species were recorded in mesic grasslands, 21 of which were exclusive to this habitat type in our sampling. Mesic grasslands also had the highest number of bee species with threatened conservation statuses, comprising 15 species: three categorised as Critically Endangered (CR), four as Endangered (EN) and eight as Vulnerable (VU). This habitat was particularly crucial for long-tongued bees from the Apidae family, including all six threatened bumblebee species in our dataset and the two Belgian species of *Eucera*, likely due to the abundance of flowering plants of the Fabaceae family. Similarly, the ‘other habitats’ category supported a substantial number of species, with 13 species exclusively found in these environments. Eleven of these were associated with slate and schist quarries, which provide suitable habitats for species typical of forested, thermophilic and/or rocky environments, such as *Osmiaparietina* and *Megachileligniseca* (Schmidt et al. 2017, Falk and Lewington 2019). The quarries which we sampled remain relatively open within large forest patches, offering nesting and floral resources that might be absent as the forest fully regenerates. Thus, maintaining this type of habitat is vital for maintaining a high bee diversity within the National Park. The wet meadow had the fewest species exclusive to it, yet harboured nearly as many threatened species (n = 11) as the tall-herb communities of humid meadows and the ‘other habitats’ (seven and nine, respectively). Nearly half of the *Trachusabyssina* population, a legally protected species in Wallonia, was also found in wet meadows, where they mainly foraged on big trefoil (*Lotuspedunculatus*). Additionally, almost all *Melittanigricans* specimens were located in wet grasslands and tall-herb communities, where their host plant, the purple loosestrife (*Lythrumsalicaria*), was abundant. Species richness across habitat types was then assessed using a generalised linear model (GLM) with a generalised Poisson distribution to address overdispersion. Statistical analyses were conducted using the R packages glmmTMB (Brooks et al. 2017) and emmeans (Lenth 2017). No significant differences in species richness were detected between habitat categories (*p* > 0.05). The lack of significant results is likely due to the limited number of sites per habitat category (n = 8 per category), which restricts the statistical power to detect differences amongst groups. Nevertheless, this finding underscores the importance of each habitat type, as they support distinct species.

Eighteen species were found across all four habitat categories, suggesting a broader ecological niche. Amongst them, *Bombussoroeensis* is Red-listed as Vulnerable (VU). This species is often misidentified in Belgium due to its close resemblance to the far more common *Bombuslapidarius*. *Bombussoroeensis* is typically associated with forest and boreal climates (Folschweiller 2020) and climate warming may pose a threat to its Belgian populations.

Finally, we identified two species that were not evaluated (NE) in the Belgian Red List (Drossart et al. 2019). The first, *Nomadabraunsiana*, is a cleptoparasitic bee previously documented by a single record in Belgium (Vertommen et al. 2024). This species is widespread across Europe, with occurrences reported in most neighbouring countries (Smit 2018). However, its host species, *Andrenacurvungula* and *Andrenapandellei*, are rare and were not detected in our sampling. Recent findings, including its discovery in the Netherlands in 2020 (Fernhout and Rhebergen 2020), suggest that *N.braunsiana* may be expanding its range. The second species with a NE Red-list status, *Andrenaafzeliella*, belongs to the Taeniandrena subgenus and was recently confirmed as part of the Belgian fauna (Wood 2023). Advances in resolving the taxonomic challenges within this subgenus facilitated its identification. A review of historical collections revealed that *A.afzeliella* has been present in Belgium for a considerable period, albeit previously unrecognised (Wood 2023). *Andrenaafzeliella* exhibits a dietary preference for flowers of the Fabaceae family and is bivoltine, with generations occurring in late spring and mid-summer (Praz et al. 2022).

## Discussion

We recorded 120 bee species during a five-month sampling period in the Semois Valley National Park (SVNP), Belgium. Of these, 22 are listed as VU, EN or CR on the Belgian Red List, representing a notably high proportion of threatened species. We identified four ecological categories of particular importance for wild bee conservation:

**Forest and Clearing Species**: A significant number of threatened species are associated with forests and clearings. Conservation measures should focus on the promotion of habitat heterogeneity by creating and maintaining early successional stages and preventing the recolonisation or abandonment of existing openings (Taki et al. 2013, Odanaka and Rehan 2020). These disturbed areas should be promoted along more preserved parcels of trees, as canopy gaps and standing dead trees in old-growth forests may support distinct bee communities (Eckerter et al. 2022).

**Fabaceae-Associated Species**: Many bee species depend on Fabaceae plants naturally found in mesic grasslands, though some Fabaceae, like *Lotuspedunculatus*, are also found in marshy areas. In degraded habitats targeted for restoration, it is essential to ensure the presence of Fabaceae species. Integrating these plants into agricultural practices, as a natural alternative to industrial fertilisers, may further support these species. Legume crop seed mixes can be optimised to include both early- and late-flowering species, enhancing support for bumblebee populations that typically exhibit extended periods of activity (Pywell et al. 2005, Cole et al. 2022).

**Thermophilic Species**: Numerous threatened species in our sample are adapted to warm, open environments, highlighting the critical role of regional quarry sites in supporting bee diversity. Effective management of these areas should focus on maintaining early-successional stages rich in floral resources, while preventing forest colonisation of exposed nesting sites (Kettermann et al. 2022). We advocate granting protection status to several slate quarries, accompanied by targeted management practices to conserve their unique ecological characteristics. Additionally, further consideration should be given to the potential role of nearby watercourses in providing similar nesting habitats.

**Wetland Community**: Wetland habitats host a unique, albeit less diverse, community including species specialised on *Lysimachia*, such as *Macropiseuropaea* and *Macropisfulvipes*, as well as their associated parasite *Epeoloidescoecutiens*. Protecting these habitats is, therefore, essential for maintaining this distinctive assemblage.

Repeated collection efforts and further observations of wild bees in the National Park of the Semois Valley are expected to reveal species that have been overlooked in the present study. Further work should ultimately allow us to compare the diversity of the Park to other protected areas in Belgium and abroad and contribute to anticipate the potential threats that these bee communities species will face in a context of global change. These results will guide the SVNP and other protected areas in Belgium in focusing conservation efforts on sites that harbour both the highest species diversity and the greatest number of threatened species.

## Figures and Tables

**Table 1.** **Table 1.** Inventory of collected species in the National Park of the Semois Valley (Belgium) in 2024. The table provides the proportion of individuals collected across the different sampled habitats, as well as the total number of specimens collected and the Red List status in Belgium. CR: Critically Endangered, EN: Endangered, VU: Vulnerable, NT: Near Threatened, LC: Least Concern, DD: Data Deficient, NE: Not Evaluated.

**Taxon**	**Conservation status in Belgium**	**Protected in Wallonia**	**Mesic grasslands**	**Tall-herb communities**	**Wet grasslands**	**Other habitats**	**Total**
**Andrenidae**							
*Andrenaafzeliella*	NE	No	0	1	0	1	2
*Andrenaangustior*	NT	No	0	0	1	2	3
*Andrenachrysosceles*	LC	No	2	0	2	0	4
*Andrenacineraria*	LC	No	5	0	1	0	6
*Andrenaclarkella*	LC	No	0	0	1	0	1
*Andrenacoitana*	EN	No	0	1	0	2	3
*Andrenadenticulata*	NT	No	1	1	1	3	6
*Andrenadorsata*	LC	No	1	1	0	0	2
*Andrenaflavipes*	LC	No	0	0	0	1	1
*Andrenafucata*	VU	No	1	2	4	1	8
*Andrenafulvago*	NT	No	3	1	0	0	4
*Andrenagravida*	LC	No	5	0	1	0	6
*Andrenahaemorrhoa*	LC	No	14	1	9	6	30
*Andrenahelvola*	VU	No	1	0	0	0	1
*Andrenahumilis*	LC	No	1	0	0	0	1
*Andrenalabialis*	NT	No	6	0	0	0	6
*Andrenalathyri*	NT	No	1	3	0	0	4
*Andrenaminutula*	LC	No	1	2	7	1	11
*Andrenanigroaenea*	LC	No	0	1	0	6	7
*Andrenanitida*	LC	No	3	0	4	0	7
*Andrenaovatula*	NT	No	3	0	4	5	12
*Andrenarosae*	LC	No	2	0	0	0	2
*Andrenaschencki*	EN	No	2	0	1	0	3
*Andrenascotica*	LC	No	1	2	2	0	5
*Andrenasubopaca*	LC	No	10	0	8	12	30
*Andrenatarsata*	EN	No	0	0	0	1	1
*Andrenavaga*	LC	No	1	0	1	0	2
*Andrenawilkella*	NT	No	13	0	8	2	23
*Panurgusbanksianus*	LC	Yes	3	0	0	2	5
*Panurguscalcaratus*	LC	Yes	4	0	0	3	7
**Apidae**							
*Anthophorafurcata*	LC	No	0	0	2	1	3
*Anthophoraplumipes*	LC	No	1	6	0	0	7
*Bombusbohemicus*	NT	No	13	1	8	6	28
*Bombuscampestris*	VU	No	2	1	2	0	5
*Bombushortorum*	NT	No	8	13	16	20	57
*Bombushumilis*	CR	Yes	1	0	0	0	1
*Bombushypnorum*	LC	No	0	1	0	1	2
*Bombuslapidarius*	LC	No	10	3	2	1	16
*Bombuslucorum*	NT	No	1	0	1	5	7
*Bombusnorvegicus*	VU	No	1	0	0	0	1
*Bombuspascuorum*	LC	No	25	69	29	44	167
*Bombuspratorum*	LC	No	2	12	7	10	31
*Bombusruderarius*	EN	No	3	0	0	0	3
*Bombusrupestris*	EN	No	1	1	0	0	2
*Bombus* spp. *sensu stricto*	LC	No	23	11	12	18	64
*Bombussoroeensis*	VU	No	7	7	5	2	21
*Bombussylvarum*	CR	Yes	4	0	1	0	5
*Bombussylvestris*	LC	No	7	1	1	4	13
*Bombusvestalis*	NT	No	1	0	0	0	1
*Ceratinacyanea*	LC	No	0	0	0	1	1
*Epeoloidescoecutiens*	LC	Yes	1	0	0	1	2
*Euceralongicornis*	VU	Yes	4	2	1	0	7
*Euceranigrescens*	EN	Yes	1	0	0	0	1
*Nomadabraunsiana*	NE	No	1	0	0	0	1
*Nomadaflava*	LC	No	1	0	1	0	2
*Nomadaflavogutta*	LC	No	2	0	2	17	21
*Nomadafucata*	LC	No	1	0	0	0	1
*Nomadafulvicornis*	LC	No	1	0	0	0	1
*Nomadagoodeniana*	LC	No	1	0	1	0	2
*Nomadaleucophthalma*	LC	No	0	1	0	0	1
*Nomadaruficornis*	LC	No	2	0	0	0	2
*Nomadarufipes*	NT	No	0	0	0	3	3
*Nomadasexfasciata*	CR	No	0	1	0	0	1
*Nomadasignata*	LC	No	2	0	0	0	2
*Nomadasuccincta*	LC	No	0	0	1	0	1
**Colletidae**							
*Colletescunicularius*	LC	Yes	1	0	0	0	1
*Colletesdaviesanus*	LC	No	2	21	9	0	32
*Hylaeuscommunis*	LC	No	0	4	0	3	7
*Hylaeusconfusus*	LC	No	2	3	1	4	10
*Hylaeusdifformis*	LC	No	0	0	3	0	3
*Hylaeusincongruus*	DD	No	0	1	3	1	5
*Hylaeusrinki*	VU	No	0	1	0	0	1
**Halictidae**							
*Halictusmaculatus*	VU	No	1	0	2	0	3
*Halictusrubicundus*	LC	No	4	3	2	3	12
*Halictusscabiosae*	LC	No	14	1	2	0	17
*Halictussexcinctus*	VU	No	5	0	1	1	7
*Halictussimplex*	EN	No	0	0	2	3	5
*Lasioglossumalbipes*	NT	No	3	0	0	0	3
*Lasioglossumcalceatum*	LC	No	10	14	10	6	40
*Lasioglossumcostulatum*	CR	No	1	0	1	1	3
*Lasioglossumlaticeps*	LC	No	0	3	0	1	4
*Lasioglossumlativentre*	LC	No	1	0	4	3	8
*Lasioglossumleucopus*	NT	No	0	1	2	6	9
*Lasioglossumleucozonium*	LC	No	6	0	0	6	12
*Lasioglossummajus*	LC	No	0	1	0	0	1
*Lasioglossummorio*	LC	No	1	2	2	2	7
*Lasioglossumpallens*	LC	No	0	0	0	1	1
*Lasioglossumparvulum*	LC	No	0	1	0	3	4
*Lasioglossumpauxillum*	LC	No	6	0	6	1	12
*Lasioglossumpunctatissimum*	LC	No	1	0	2	2	5
*Lasioglossumrufitarse*	NT	No	0	1	0	1	2
*Lasioglossumsexnotatum*	LC	No	0	11	3	2	16
*Lasioglossumvillosulum*	LC	No	6	0	2	17	25
*Lasioglossumzonulum*	LC	No	5	3	6	0	14
*Seladoniatumulorum*	LC	No	2	0	2	2	6
*Sphecodescrassus*	LC	No	0	0	0	1	1
*Sphecodesephippius*	LC	No	3	4	2	6	15
*Sphecodesgeoffrellus*	LC	No	0	0	1	0	1
*Sphecodesmonilicornis*	LC	No	1	0	0	0	1
*Sphecodespuncticeps*	LC	No	1	0	0	0	1
*Sphecodesreticulatus*	LC	No	0	0	0	1	1
**Megachilidae**							
*Anthidiellumstrigatum*	LC	No	1	0	0	1	2
*Anthidiumoblongatum*	LC	No	1	0	0	0	1
*Chelostomaflorisomne*	LC	No	4	3	1	0	8
*Heriadestruncorum*	LC	No	0	1	0	0	1
*Hoplitisadunca*	LC	No	0	0	0	10	10
*Hoplitisclaviventris*	VU	No	0	0	0	1	1
*Megachilelapponica*	LC	No	0	0	0	1	1
*Megachileligniseca*	LC	No	0	0	0	1	1
*Megachileversicolor*	LC	No	0	0	0	1	1
*Megachilewillughbiella*	LC	No	0	1	1	0	2
*Osmiabicolor*	LC	Yes	0	0	0	1	1
*Osmiabicornis*	LC	No	4	0	0	0	4
*Osmialeaiana*	LC	No	5	0	2	3	10
*Osmiaparietina*	EN	No	0	0	0	1	1
*Trachusabyssina*	LC	Yes	20	1	19	7	47
**Melittidae**							
*Macropiseuropaea*	LC	Yes	1	9	5	0	15
*Macropisfulvipes*	LC	Yes	0	3	2	0	5
*Melittahaemorrhoidalis*	LC	No	1	0	0	0	1
*Melittanigricans*	LC	No	2	8	10	0	20
